# The discussion of risk in German surgical clinical practice guidelines: a qualitative review

**DOI:** 10.1515/iss-2020-0026

**Published:** 2021-08-25

**Authors:** Stuart McLennan, Carolin Jansen, Alena Buyx

**Affiliations:** Institute of History and Ethics in Medicine, TUM School of Medicine, Technical University of Munich, Munich, Germany; Fachbereich Medizinethik, Institut für Experimentelle Medizin, Christian-Albrechts-Universität zu Kiel, Kiel, Germany

**Keywords:** clinical practice guideline, risks, surgery

## Abstract

**Objectives:**

Clinical practice guidelines (CPGs) have a potentially important role regarding the assessment and communication of the risks of perioperative complications. This study aimed to (1) examine the content of German surgical CPGs in relation to surgical risks and (2) provide baseline results for future research in order to assess the development of surgical CPGs in Germany in relation to this issue.

**Methods:**

In November 2015, all German surgical CPGs that provide guidance regarding illnesses that can be treated with a surgical procedure were collected from the websites of the German umbrella organisation of medical professional associations and the German Association for Cardiology.

**Results:**

Data collection retrieved 230 CPGs of which 214 were included in the final analysis. The analysis identified four different groups: 1) 5% (10/214) of guidelines did not discuss “risks” or “complications” at all; 2) 21% (44/214) of guidelines discussed general risks that are not related to surgical complications; 3) 35% (76/214) of guidelines discussed surgical complications and often discussed their likelihood in terms of “high risk” or “low risk”, but did not provide numeric estimates and 4) 39% (84/214) of guidelines discussed specific surgical risks and also provided numerical risk estimates. Guidelines with higher methodological quality more frequently included numerical risk estimates.

**Conclusions:**

It is positive that the vast majority of German surgical CPGs address the issue of risks. However, it would be helpful if more German surgical CPGs provide explicit and evidence-based estimates and recommendations relating to the surgical risk to support surgeons in providing high-quality care and to meet their ethical obligations to patients.

## Introduction

Perioperative complications are significant sources of morbidity and mortality [1], [2], [3], [4]. A recent analysis suggests that postoperative mortality presents the third leading cause of death; with at least 4.2 million people worldwide dying within 30 days of surgery each year [5]. In this context, appropriate assessment and communication regarding the risk of perioperative complications are important to enable individual care to be optimised. In addition, respecting patient autonomy requires clear communication so that patients can be provided with comprehensive information about risks and can make informed decisions concerning their treatment [6].

Evidence-based medicine emerged in the 20th century stressing the importance of integrating clinical expertise with the best available evidence [7]. As part of this movement, the importance of processed evidence was recognised. Clinical practice guidelines (CPGs) are now widely used to help standardise clinical practice and assist physician and patient decisions about appropriate health care for specific clinical circumstances [8, 9]. Given there is also an expectation in most countries that physicians follow CPGs, surgical CPGs have a potentially important role regarding not only the assessment but also the communication of surgery-related risks.

However, we are not aware of any previous analysis of whether and how surgical CPGs’ recommendations deal with surgery-related risks. An empirical analysis of surgical CPGs in relation to this issue may help identify gaps and areas in which GCPs could be developed in order to provide better guidance, for example on how to inform patients. This study, therefore, aimed to (1) examine the content of German surgical CPGs in relation to surgical risks and (2) provide baseline results for future research in order to assess the development of surgical CPGs in Germany in relation to this issue.

## Methods

### Inclusion criteria

To be included in the review, a CPG had to:

1) Provide guidance regarding illnesses that can be treated with a surgical procedure. This includes CPGs published by surgical associations as well as non-surgical associations. A surgical procedure was defined as “any invasive clinical intervention that causes a transient or permanent alteration to the human body”, this includes incision, excision, resection, puncture, biopsy, amputation, local destruction or modification of tissues or organs, implantation of biological or synthetic materials and suturing or stapling.
2) Be in force in Germany in November 2015.

### Data collection

The German umbrella organisation of medical professional associations has the most comprehensive database of German CPGs and was therefore selected as the main database for data collection. However, the German Association for Cardiology (DGK) publishes their own CPGs on invasive cardiology procedures independently on their own website. It appears that the DGK’s process for developing GPGs is similar to other specialities. In November 2015, all CPGs publicly available on the websites were collected by Carolin Jansen (CJ). Based on the inclusion criteria, CJ screened the CPGs in order to assess for eligibility for inclusion in the review. In case of uncertainty, the CPG was discussed with project members.

### Data analysis

Using the included CPGs, CJ performed conventional content analysis [10]. Initial themes were identified inductively using a process of open coding (i.e. no specific preconceived codes were identified or used; rather, codes emerged directly from the data). A coding framework was developed by a progressive process of classifying, comparing and refining text passages to create categories. The final coding framework was checked by Stuart McLennan and Alena Buyx to ensure consistency and validity.

## Results

Data collection retrieved 230 CPGs of which 214 were included in the final analysis. The included CPGs were published by a total of 11 different specialities (see Table 1), however, 73.3% (157/214) of included GPGs came from fives specialities (paediatric surgery, neurosurgery, trauma and orthopaedic surgery, vascular surgery and general and visceral surgery). Overall, 36.9% (79/214) of included CPGs had the methodological quality of S1 “Recommendations by committee of experts”, 37.8% (81/214) had one of the methodological qualities of S2/S2e/S2k “Guidelines based on evidence or consensus of a representative committee” and 18.2% (39/214) had the methodological quality of S3 “Guidelines based on evidence and consensus of a representative committee” (see Table 2).

**Table 1:** Summary of guidelines and specialities.

Specialty	Total guidelines	Risk group			
	n/N, %	No discussion of risks n/N, %	General risks n/N, %	Specific risks without numeric estimates n/N, %	Specific risks with numeric estimates n/N, %
Paediatric surgery	58/214	6/10	17/44	15/76	20/84 (23.8)
	(27.1)	(60)	(39)	(19.7)	
Neurosurgery, including invasive neurology	29/214 (13.5)	1/10 (10)	10/44 (23)	10/76	8/84
				(13.2)	(9.5)
Trauma and orthopaedic surgery	26/214 (12.1)	0	1/44 (2)	4/76 (5.3)	21/84 (25)
Vascular surgery	25/214 (11.7)	0	8/44 (18)	9/76 (11.8)	8/84 (9.5)
General and visceral surgery	19/214 (8.9)	1/10 (10)	1/44 (2)	13/76 (17.1)	4/84 (4.8)
Obstetrics and gynaecology	15/214 (7.0)	1/10 (10)	1/44 (2)	9/76 (11.8)	4/84 (4.8)
Interdisciplinary guidelines & collaborations	14/214 (6.5)	0	0	12/76 (15.8)	2/84 (2.4)
Cardiothoracic surgery, including invasive cardiology	13/214 (6.1)	0	2/44 (5)	9/76 (11.8)	2/84 (2.4)
Ear nose throat	8/214 (3.7)	0	0	2/76 (2.6)	6/84 (7.1)
Dermatology	6/214 (2.8)	1/10 (10)	4/44 (9)	0	1/84 (1.2)
Urology	1/214 (0.5)	0	0	1/76 (1.3)	0

**Table 2:** Summary of guidelines and quality category.

Quality category^a^	Total guidelines n/N, %	Risk group			
		No discussion of risks n/N, %	General risks n/N, %	Specific risks without numeric estimates n/N, %	Specific risks with numeric estimates n/N, %
S1	79/214	9/10	20/44	31/76	19/84
	(36.9)	(90)	(45.5)	(40.8)	(22.6)
S2	24/214	1/10	10/44	7/76	6/84
	(11.2)	(10)	(22.7)	(9.2)	(7.1)
S2e	9/214	0/10	1/44	6/76	2/84
	(4.2)		(2.3)	(7.9)	(2.4)
S2k	48/214	0/10	10/44	22/76	16/84
	(22.4)		(22.7)	(28.9)	(19)
S3	39/214	0/10	1/44	8/76	30/84
	(18.2)		(2.3)	(10.5)	(35.7)
N/A	15/214	0/10	2/44	2/76	11/84
	(7)		(4.5)	(2.6)	(13.1)

^a^S1: Recommendations by committee of experts; S2: Guidelines based on evidence (S2e) or consensus of a representative committee (S2k); S3: Guidelines based on evidence and consensus of a representative committee.

Analysis of the 214 CPGs identified four different risk groups:

**(1) No discussion of risks:** 5% (10/214) of guidelines did not discuss “risks” or “complications” at all. The majority of guidelines in this group came from paediatric surgery (6/10; 60%) and had the methodological quality of S1 (9/10; 90%).
**(2) General risks:** 21% (44/214) of guidelines discussed general risks that are not related to surgical complications (e.g. genetic predisposition). The majority (35/44; 79.5%) of guidelines in this group were from paediatric surgery (17/44), neurosurgery (10/44) or vascular surgery (8/44). Most guidelines in this group had a methodological quality of S2/S2e/S2k (21/44; 47.7%) or S1 (20/44; 44.5%).
**(3) Specific risks without numeric estimates:** 35% (76/214) of guidelines discussed surgical complications and often discussed their likelihood in terms of “high risk” or “low risk”, but did not provide numeric estimates. Over a half of the guidelines (41/76; 53.9%) in this group were from trauma and orthopaedic surgery (21/76) and paediatric surgery (20/76). Most guidelines in this group had a methodological quality of S2/S2e/S2k (35/76; 46.1%) or S1 (31/76; 40.8%).
**(4) Specific risks with numeric estimates:** 39% (84/214) of guidelines discussed specific surgical risks and also provided numerical risk estimates. These guidelines were spread across a number of specialities, but just under half of them (41/84; 48.8%) came from paediatric surgery (20/84) and trauma and orthopaedic surgery (21/84). The guidelines in this group were also spread across methodological quality; 35.7% (30/84) were S3, 28.6% (24/84) were S2/S2e/S2k and 22.6% (19/84) were S1.

Supplementary Table 1 lists all included CPGs by risk group, including CPG title, methodological quality category, author and year updated.

## Discussion

To the best of our knowledge, this is the first study to assess a comprehensive national sample of surgical CPGs in relation to surgery-related risks. In a field that routinely deals with interventions that can have significant risks for patients, it is positive that the vast majority of German surgical CPGs address the issue of risks. However, despite the prevalence of risk assessment tools, it appears that risk assessment has not yet adequately penetrated surgical CPGs in Germany. The lack of explicit and evidence-based risk estimates and recommendations in many CPGs is ethically problematic.

Although there has been progress in using the best available evidence to improve clinical decision-making, there remains significant unwarranted variation among treatments that clinicians and health systems routinely use in practice and deficiencies regarding all key aspects of health care [11]. The insufficient protection of patients from unjustified harms and burdens from clinical care has been identified as a “profoundly serious moral problem” [12]. The absence of clear risk assessment and communication recommendations in CPGs is likely to exacerbate unwarranted variability in treatment [13], as clinical intuition can often disregard critical data and be influenced by cognitive biases [14]. Choosing an ill-suited surgical approach for patients that leads to complications also has wider ethical implications than just the harm they can cause to patients. Most complications involve caring and competent clinicians, who can be emotionally or psychologically harmed as a result of the complication [15]. Empirical evidence suggests that clinicians involved in major complications, without sufficient support, can experience burn-out, difficulty sleeping, depression, flashbacks and self-doubt; harming not only their health but also threatening their ability to deliver safe, compassionate care [16], [17], [18], [19], [20]. In circumstances where revision surgery is required, limited health resources can be wasted and delays caused to other planned procedures, thus negatively affecting other patients [21]. Furthermore, insufficient information about the risk of procedures can undermine patient´s right to make informed decisions concerning their treatment. This could run against the established ethical principle of respecting patient autonomy [6].

Objective criteria for clinical decision-making are considered an essential part of evidence-based surgery [22]. Although exceptions may apply where CGPs cover rare conditions and available evidence does not yet support risk scoring or stratification, CPGs are an important source of well-structured, current information for clinical use that can help surgeons to select the most suitable treatment for patients. They are also a tool that can make it easier to communicate with the patient in an appropriate manner. From an ethical point of view, a strong case can be made that all CPGs in surgery should include clear information and treatment recommendations based on an in-depth consideration of specific, procedure-related risks. Obviously, many factors need to guide clinical decision-making. However, guideline creators in medical associations should be encouraged to include more risk assessments and risk estimates in their guidelines and discuss these more explicitly, for the benefit of all actors involved.

### Limitations

This review has some limitations that should be taken into account when interpreting the results. First, the only CPGs from Germany were included in the review. Risk assessment in CPGs in other countries might differ significantly from our sample. However, the finding that a substantial number of German CPGs do not provide specific guidance regarding the risk assessment of surgical complications can help highlight the importance of this issue internationally. Second, it should be kept in mind that oftentimes international guidelines are widely used in clinical practice in Germany and therefore no national guideline exists; the guideline sample used in this analysis is by no means a complete depiction of all surgical procedures performed in hospitals in the country. Finally, CPGs were collected in November 2015 and a number of these may have expired or undergone review. However, we do not believe the fact that some of the analysed CPGs may now be out of date significantly undermines our review. This review has utilised a cross-sectional approach, analysing German CPGs at a specific point in time (November 2015). This provides baseline results for future research in order to assess the development of German CPGs in relation to surgical complications.
