# Supplementary material for: The discussion of risk in German surgical clinical practice guidelines: a qualitative review
Source: Innov Surg Sci. 2021 Aug 25;6(2):53–7. doi: 10.1515/iss-2020-0026 (PMC8435268; doi:10.1515/iss-2020-0026)
Supplement: Supplementary file 1 [file iss-06-20200026-s001.docx]

## Supplementary **Table 1. Included CGPs**

| **Nr.** | **CGP Title** | **Quality**  **Category^1^** | **Author** | **Last Updated** |
| --- | --- | --- | --- | --- |
| **No discussion of risks (n=10)** | | | | |
| 1 | Anal rim carcinoma  [Analrand-Karzinom] | S1 | German Society for Coloproctology | 2002 |
| 2 | Invagination  [Invagination] | S1 | German Society for Paediatric Surgery | 2013 |
| 3 | Diaphragmatic hernia, diaphragmatic defect  [Zwerchfellhernie, Zwerchfelldefekt] | S1 | German Society for Paediatric Surgery | 2010 |
| 4 | Aortic valve insufficiency in children and adolescents  [Aortenklappeninsuffizienz bei Kindern und Jugendlichen] | S2 | German Society for Paediatric Cardiology | 2013 |
| 5 | High-grade malignant gliomas and ponsgliomas  [Hochgradig maligne Gliome und Ponsgliome] | S1 | Society for Paediatric Oncology and Haematology | 2008 |
| 6 | Craniopharyngeomas in childhood and adolescence  [Kraniopharyngeome im Kindes- und Jugendalter] | S1 | Society for Paediatric Oncology and Haematology | 2013 |
| 7 | Ependymoma  [Ependymome] | S1 | Society for Paediatric Oncology and Haematology | 2010 |
| 8 | Diagnosis and therapy of trophoblastic tumours  [Diagnostik und Therapie der Trophoblasttumore] | S1 | German Cancer Society and German Society for Gynaecology & Obstetrics | 2008 |
| 9 | Microscopically controlled surgery  [Mikroskopisch kontrollierte Chirurgie (MKC)] | S1 | German Dermatological Society | 2014 |
| 10 | Cervical radiculopathy  [Zervikale Radikulopathie] | S1 | German Society for Neurology | 2012 |
| **General Risks (n=44)** | | | | |
| 11 | Guideline Angiosarcoma of the skin and Kaposi's sarcoma  [Leitlinie Angiosarkom der Haut und Kaposi-Sarkom] | S1 | German Cancer Society and German Dermatological Society | 2012 |
| 12 | Dermatofibrosarcoma protuberans  [Dermatofibrosarkoma protuberans] | S1 | German Cancer Society and German Dermatological Society | 2012 |
| 13 | Extracranial germ cell tumours [Extrakranielle Keimzelltumoren] | S1 | Society for Paediatric Oncology and Haematology | 2012 |
| 14 | Anal genital warts  [Anale Feigwarzen] | S1 | German Society for Coloproctology | 2008 |
| 15 | Traumatic rupture of the spleen in childhood  [Traumatische Milzruptur im Kindesalter] | S1 | German Society for Paediatric Surgery | 2015 |
| 16 | Guidelines for the implantation of defibrillators  [Leitlinien zur Implantation von Defibrillatoren] | N/A | Jung et al. | 2006 |
| 17 | Acute intestinal artery occlusion  [Akuter Intestinalarterienverschluss] | S2 | German Society for Vascular Surgery and Vascular Medicine | 2011 |
| 18 | Acute peripheral arterial occlusion  [Der akute periphere Arterienverschluss] | S2 | German Society for Vascular Surgery and Vascular Medicine | 2011 |
| 19 | Acute therapy of ischaemic stroke  [Akuttherapie des ischämischen Schlaganfalls] | S1 | German Society for Neurology | 2012 |
| 20 | Guideline on the limb at risk of amputation  [Leitlinie zur amputationsbedrohten Extremität] | S2 | German Society for Vascular Surgery and Vascular Medicine | 2008 |
| 21 | Acceleration trauma of the cervical spine  [Beschleunigungstrauma der Halswirbelsäule] | S1 | German Society for Neurology | 2012 |
| 22 | Treatment of Blitz-Nick-Salaam Epilepsy  [Therapie der Blitz-Nick-Salaam Epilepsie] | S3 | German Society for Neuropaediatrics | 2009 |
| 23 | Ebstein anomaly of the tricuspid valve in children and adolescents  [Ebstein-Anomalie der Trikuspidalklappe bei Kindern und Jugendlichen] | S2 | German Society for Paediatric Cardiology | 2014 |
| 24 | Guideline on endangiitis obliterans  [Leitlinie zur Endangiitis obliterans] | S2 | German Society for Vascular Surgery and Vascular Medicine | 2011 |
| 25 | Ewing sarcomas in children and adolescents  [Ewing-Sarkome im Kinder- und Jugendalter] | S1 | Society for Paediatric Oncology and Haematology | 2014 |
| 26 | Fallot'sche Tetralogie im Kindes- und Jugendalter  [Fallot'sche Tetralogie im Kindes- und Jugendalter] | S2 | German Society for Paediatric Cardiology | 2013 |
| 27 | Haemangiomas in infancy and early childhood  [Hämangiome im Säuglings- und Kleinkindesalter] | S2k | German Society for Paediatric Surgery | 2012 |
| 28 | Hepatoblastoma  [Hepatoblastom] | S1 | Society for Paediatric Oncology and Haematology | 2010 |
| 29 | Brain abscess  [Hirnabszess] | S1 | German Society for Neurology | 2012 |
| 30 | Guideline invasive electrophysiological diagnostics  [Leitlinie invasive elektrophysiologische Diagnostik] | N/A | Willems et al. | 2007 |
| 31 | Diagnosis and therapy of postthrombotic syndrome  [Diagnostik und Therapie des Postthrombotischen Syndroms] | S2 | German Society for Vascular Surgery and Vascular Medicine | 2008 |
| 32 | Merkel cell carcinoma  [Merkelzellkarzinom] | S2k | German Cancer Society and German Dermatological Society | 2012 |
| 33 | Establishment of arteriovenous vascular accesses for haemodialysis as well as for diagnosis and therapy of access-associated complications  [Anlage von arteriovenösen Gefäßzugängen zur Hämodialyse sowie zur Diagnostik und Therapie von Zugangs-assoziierten Komplikationen] | S2 | Deutsche Gesellschaft für Gefäßchirurgie und Gefäßmedizin | 2008 |
| 34 | Thermal injuries in childhood  [Thermische Verletzungen im Kindesalter] | S2k | German Society for Vascular Surgery and Vascular Medicine | 2009 |
| 35 | Basal cell carcinoma of the skin  [Basalzellkarzinom der Haut] | S2k | German Cancer Society and German Dermatological Society | 2013 |
| 36 | Unterarmschaftfraktur im Kindesalter  [Unterarmschaftfraktur im Kindesalter] | S1 | German Society for Paediatric Surgery | 2013 |
| 37 | Diagnosis and treatment of stenoses of the vertebral arteries  [Diagnostik und Behandlung von Stenosen der Vertebralarterien] | S2 | German Society for Vascular Surgery and Vascular Medicine | 2011 |
| 38 | Cerebral Sinus & Vein Thrombosis  [Zerebrale Sinus- & Venenthrombose] | S1 | German Society for Neurology | 2012 |
| 39 | Cerebral vasculitis  [Zerebrale Vaskulitis] | S1 | German Society for Neurology | 2012 |
| 40 | Cervical spondylotic myelopathy  [Zervikale spondylotische Myelopathie] | S1 | German Society for Neurology | 2012 |
| 41 | Idiopathic scoliosis of growing age  [Idiopathische Skoliose im Wachstumsalter] | S1 | Gesellschaft für Orthopädie und Orthopädische Chirurgie | 2009 |
| 42 | Paraplegia  [Querschnittslähmung] | S1 | German Society for Neurology | 2012 |
| 43 | Nephroblastoma  [Nephroblastom] | S1 | Society for Paediatric Oncology and Haematology | 2010 |
| 44 | Uterine sarcomas  [Uterine Sarkome] | S2k | German Society for Gynaecology and Obstetrics | 2015 |
| 45 | Traumatic brain injury in adulthood  [Schädel-Hirn-Trauma im Erwachsenenalter] | S2e | German Society for Neurosurgery | 2007 |
| 46 | Chronic heart failure children  [Chronische Herzinsuffizienz Kinder] | S2k | Society for Paediatric Cardiology | 2015 |
| 47 | Diseases of the popliteal artery  [Erkrankungen der A. poplitea] | S2 | German Society for Vascular Surgery | 2008 |
| 48 | Lung malformations  [Lungenfehlbildungen] | S1 | German Society for Paediatric Surgery | 2015 |
| 49 | Necrotising enterocolitis  [Nekrotisierende Enterokolitis] | S2k | German Society for Neonatology and Paediatric Intensive Care Medicine | 2010 |
| 50 | Dissection of brain-supplying supra-aortic arteries  [Dissektion hirnversorgender supraaortaler Arterien] | S1 | German Society for Neurology | 2008 |
| 51 | Subarachnoid haemorrhage  [Subarachnoidalblutung] | S1 | German Society for Neurology | 2012 |
| 52 | Primary cardiomyopathies in childhood and adolescence  [Primäre Kardiomyopathien im Kindes- und Jugendalter] | S2k | German Society for Paediatric Cardiology | 2013 |
| 53 | Subvalvular aortic stenosis in children and adolescents  [Subvalvuläre Aortenstenose bei Kindern und Jugendlichen] | S2k | German Society for Paediatric Cardiology | 2013 |
| 54 | Pulmonary insufficiency  [Pulmonalinsuffizienz] | S2k | German Society for Paediatric Cardiology | 2015 |
| **Specific risks without numeric estimates (n=76)** | | | | |
| 55 | Lumbar disc herniation  [Lumbaler Bandscheibenvorfall] | S3 | German Society for Neurosurgery | 2006 |
| 56 | Epicondylopathy radialis humeri  [Epicondylopathia radialis humeri] | S1 | German Society for Orthopaedics and Orthopaedic Surgery | 2011 |
| 57 | Hallux valgus  [Hallux valgus] | S2e | German Society for Orthopaedics and Orthopaedic Surgery | 2015 |
| 58 | Proximal humerus fracture in children  [Proximale Humerusfraktur beim Kind] | S1 | German Society for Paediatric Surgery | 2013 |
| 59 | Active, implantable hearing systems for hearing disorders [Aktive, implantierbare Hörsysteme bei Hörstörungen] | S1 | German Society for Otorhinolaryngology and Head and Neck Surgery | 2010 |
| 60 | Congenital clubfoot  [Kongenitaler Klumpfuß] | S1 | German Society for Orthopaedics and Orthopaedic Surgery | 2012 |
| 61 | Snoring  [Schnarchen] | S2k | Working Group on Sleep Medicine of the German Society for Ear, Nose and Throat Medicine | 2013 |
| 62 | Tibia and tibia shaft fracture in childhood  [Tibia- und Unterschenkelschaftfraktur im Kindesalter] | S1 | German Society for Paediatric Surgery | 2015 |
| 63 | Bacterial joint infections [Bakterielle Gelenkinfektionen] | S1 | German Society for Trauma Surgery | 2008 |
| 64 | Abdominal wall defects [Bauchwanddefekte] | S1 | German Society for Paediatric Surgery | 2012 |
| 65 | Gastroesophageal reflux children [Gastroösophagealer Reflux Kinder] | S1 | German Society for Paediatric Surgery | 2015 |
| 66 | Patella fracture  [Patellafraktur] | S1 | German Society for Trauma Surgery | 2008 |
| 67 | Hip prosthesis replacement [Prothesenwechsel Hüfte] | S1 | German Society for Trauma Surgery | 2008 |
| 68 | Shoulder joint first dislocation [Schultergelenk-Erstluxation] | S1 | German Society for Trauma Surgery | 2009 |
| 69 | Meniscus diseases [Meniskuserkrankungen] | S1 | German Society for Orthopaedics and Orthopaedic Surgery | 2002 |
| 70 | Pertrochanteric femur fracture [Pertrochantere Oberschenkelfraktur] | S2e | German Society for Trauma Surgery | 2015 |
| 71 | Recurrent shoulder dislocation [Schulterluxation rezidivierend] | S1 | German Society for Orthopaedics and Orthopaedic Surgery | 2009 |
| 72 | Distal radius fracture  [Distale Radiusfraktur] | S2e | German Society for Trauma Surgery | 2015 |
| 73 | Seromukotympanum [Seromukotympanum] | S1 | German Society for Otorhinolaryngology, Head and Neck Surgery | 2011 |
| 74 | Squamous cell carcinoma [Plattenepithel-Carcinom] | S2k | German Cancer Society and German Dermatological Society | 2013 |
| 75 | Cholesteatoma  [Cholesteatom] | S1 | German Society for Otorhinolaryngology, Head and Neck Surgery | 2014 |
| 76 | Surgery-related injuries of the urethra in gynaecology and obstetrics  [Operationsbedingte Verletzungen des Urethers in der Gynäkologie und Geburtshilfe] | S1 | German Society for Gynaecology and Obstetrics | 2013 |
| 77 | Obstructive sialadenitis [Obstruktive Sialadenitis] | S2k | German Society for Otorhinolaryngology, Head and Neck Surgery | 2013 |
| 78 | Therapy of obstructive sleep apnoea of the Adult  [Therapie der obstruktiven Schlafapnoe des Erwachsenen] | S2 | German Society for Otorhinolaryngology, Head and Neck Surgery | 2009 |
| 79 | Medulloblastoma [Medulloblastom] | S1 | German Society for Paediatric Oncology | 2012 |
| 80 | Patella luxation  [Patella-Luxation] | S1 | German Society for Trauma Surgery | 2014 |
| 81 | Intra-articular fracture of the distal humerus in childhood [Intraartikuläre Fraktur des distalen Humerus im Kindesalter] | S2k | German Society for Paediatric Surgery | 2015 |
| 82 | Anorectal malformations [Anorektale Fehlbildungen] | S1 | German Society for Paediatric Surgery | 2013 |
| 83 | Aortic valve stenosis children [Aortenklappenstenose Kinder] | S2k | German Society for Paediatric Cardiology | 2013 |
| 84 | Atrioventricular septal defect [Atrioventrikulärer Septumdefekt] | S2k | German Society for Paediatric Cardiology | 2013 |
| 85 | Chronic occlusive processes of the intestinal arteries  [Chronische Verschlussprozesse der Intestinalarterien] | S2 | German Society for Vascular Surgery and Vascular Medicine | 2008 |
| 86 | Double Outlet Right Ventricle | S2k | German Society for Paediatric Cardiology | 2013 |
| 87 | Femoral shaft fracture child  [Femurschaftfraktur Kind] | S1 | German Society for Trauma Surgery | 2014 |
| 88 | Vascular Injuries  [Gefäßverletzungen] | S2 | German Society for Vascular Medicine and Vascular Surgery | 2008 |
| 89 | Glioma  [Gliome] | S2k | German Society of Neurology | 2014 |
| 90 | Cardiac pacemaker  [Herzschrittmacher] | N/A | German Society for Cardiology, Heart and Circulation Research | 2005 |
| 91 | Brain metastases  [Hirnmetastasen] | S2k | German Society of Neurology | 2014 |
| 92 | Implant removal  [Implantatentfernung] | S1 | German Society for Trauma Surgery | 2013 |
| 93 | Intracranial pressure  [Intrakranieller Druck] | S1 | German Society of Neurology | 2012 |
| 94 | Intracerebral haemorrhage  [Intrazerebrale Blutung] | S2e | German Society of Neurology | 2012 |
| 95 | Invasive electrophysiological diagnostics  [Invasive elektrophysiologische Diagnostik] | S3 | German Society for Cardiology, Heart and Circulation Research | 2007 |
| 96 | Germ cell tumours  [Keimzelltumoren ZNS] | S1 | Society for Paediatric Oncology and Haematology | 2012 |
| 97 | Surgical Therapy of Benign Thyroid Diseases  [Operative Therapie benigner Schilddrüsenerkrankungen] | S2k | German Society for General and Visceral Surgery, Surgical Working Group on Endocrinology | 2010 |
| 98 | Neuroblastoma  [Neuroblastom] | S1 | Society for Paediatric Oncology and Haematology | 2011 |
| 99 | Short esophageal atresia  [Kurzstreckige Ösophasgusatresie] | S2k | German Society for Paediatric Surgery | 2012 |
| 100 | Abdominal aortic / iliac artery occlusion  [Bauchaorten- / Beckenarterienverschlüsse] | S2 | German Society for Vascular Medicine and Vascular Surgery | 2008 |
| 101 | Persistent ductus arteriosus  [Persistierender Ductus Arteriosus] | S2 | German Society for Paediatric Cardiology | 2010 |
| 102 | Proximal femoral fractures of the child  [Proximale Femurfrakturen des Kindes] | S2e | German Society for Trauma Surgery | 2008 |
| 103 | Pulmonary atresia with ventricular septal defect (PA-VSD) in children and adolescents  [Pulmonalatresie mit Ventrikelseptumdefekt (PA-VSD) im Kindes- und Jugendalter] | S2k | German Society for Paediatric Cardiology | 2013 |
| 104 | Supravalvular aortic stenosis in children and adolescents  [Supravalvuläre Aortenstenose im Kindes- und Jugendalter] | S2k | German Society for Paediatric Cardiology | 2013 |
| 105 | Thoracic Outlet Syndrome  [Thoracic Outlet Syndrome] | S2 | German Society for Vascular Medicine and Vascular Surgery | 2008 |
| 106 | Ulcus cruris  [Ulcus cruris] | S3 | German Society for Phlebology | 2008 |
| 107 | Lower leg artery occlusion  [Unterschenkelarterienverschlüsse] | S2 | German Society for Vascular Medicine and Vascular Surgery | 2008 |
| 108 | Ventral instability shoulder  [Ventrale Instabilität Schulter] | S1 | German Trauma Society | 2009 |
| 109 | Anterior cruciate ligament rupture  [Vordere Kreuzbandruptur] | S1 | German Trauma Society | 2008 |
| 110 | Cubital tunnel syndrome  [Kubitaltunnelsyndrom] | S3 | German Society for Neurosurgery, German Society for Neurology, German Society for Orthopaedic Surgery | 2008 |
| 111 | Knee joint near leg axis malposition  [Kniegelenksnahe Beinachsenfehlstellung] | S1 | German Society for Orthopaedics and Orthopaedic Surgery | 2009 |
| 112 | LASH | S1 | German Society of Gynaecology and Obstetrics | 2008 |
| 113 | Malignant ovarian tumours  [Maligne Ovarialtumore] | S3 | Guideline Programme Oncology | 2013 |
| 114 | Mamma-Ca | S3 | Guideline Programme Oncology | 2012 |
| 115 | Malignant thyroid diseases  [Maligne Schilddrüsenerkrankungen] | S2k | German Society for General and Visceral Surgery | 2012 |
| 116 | NASH | S2k | German Society for General and Visceral Surgery | 2015 |
| 117 | [Supracondylar humerus fracture child ]  Suprakondyläre Humerusfraktur Kind | S1 | German Trauma Society | 2014 |
| 118 | Peripheral pulmonary artery stenosis  [Periphere Pulmonalarterienstenosen] | S2k | German Society for Paediatric Cardiology | 2015 |
| 119 | Shape disorders of the inner and/or outer nose  [Formstörungen der inneren und/oder äußeren Nase] | S2k | German Society for Otorhinolaryngology, Head and Neck Surgery | 2010 |
| 120 | Anal abscess [Analabszess] | S3 | Ommer et. al. | 2011 |
| 121 | Vaginal surgical deliveries  [Vaginal operative Entbindungen] | S1 | German Society of Gynaecology and Obstetrics | 2012 |
| 122 | Acute scrotum  [Akutes Skrotum] | S2k | German Society for Paediatric Surgery, German Society for Urology | 2012 |
| 123 | Carpal tunnel syndrome  [Karpaltunnelsyndrom] | S3 | German Societies for Hand Surgery, Neurosurgery,  Neurology and Orthopaedics | 2012 |
| 124 | Hypoplastic left heart syndrome  [Hypoplastisches Linksherzsyndrom] | S2k | German Society for Paediatric Cardiology | 2013 |
| 125 | Atrial septal defect (ASD) in childhood and adolescence  [Vorhofseptumdefekt (ASD) im Kindes- und Jugendalter] | S2k | German Society for Paediatric Cardiology | 2013 |
| 126 | Femoral neck fracture in adults  [Schenkelhalsfraktur des Erwachsenen] | S2e | German Society for Trauma Surgery and Austrian Society for Trauma Surgery | 2008 |
| 127 | Endometrial carcinoma  [Endometriumkarzinom] | S2k | German Cancer Society, German Society for Gynaecology and Obstetrics | 2008 |
| 128 | Endoprosthesis in gonarthrosis  [Endoprothese bei Gonarthrose] | S1 | German Trauma Society, Austrian Trauma Society | 2009 |
| 129 | Stenoses and occlusions of the aortic arch  [Abgangsnahe Stenosen und Verschlüsse der Aortenbogenäste] | N/A | German Society for Vascular Surgery | 2008 |
| 130 | Cochlear implant  [Cochleaimplantat] | S2k | German Society for Otorhinolaryngology, Head and Neck Surgery | 2012 |
| **Specific risks with numeric estimates (n=84)** | | | | |
| 131 | Haemorrhoidal disease  [Hämorrhoidalleiden] | S1 | German Society for Coloproctology | 2008 |
| 132 | Inguinal hernia, hydrocele  [Leistenhernie, Hydrozele] | S1 | German Society for Paediatric Surgery | 2010 |
| 133 | Hypertrophic pyloric stenosis  [Hypertrophe Pylorusstenose] | S1 | German Society for Paediatric Surgery | 2013 |
| 134 | Sinus pilonidalis  [Sinus pilonidalis] | S3 | German Society for Coloproctology | 2014 |
| 135 | Adenoid vegetations  [Adenoide Vegetationen] | S1 | German Society for Otorhinolaryngology, Head and Neck Surgery | 2011 |
| 136 | Recommendations for the prevention of position-related damage in surgical gynaecology  [Empfehlungen zur Verhinderung lagerungsbedingter Schäden in der operativen Gynäkologie] | S1 | German Society for Gynaecology and Obstetrics | 2015 |
| 137 | Carpal tunnel syndrome  [Karpaltunnelsyndrom] | S3 | German Society for Neurosurgery, German Society for Neurology, German Society for Orthopaedics and Orthopaedic Surgery | 2012 |
| 138 | Hirschsprung's disease  [M. Hirschsprung] | S1 | German Society for Paediatric Surgery | 2013 |
| 139 | Ankle fracture  [Sprunggelenksfraktur] | S1 | German Society for Trauma Surgery | 2008 |
| 140 | Truncus arteriosus communis  [Truncus arteriosus communis] | S2k | German Society for Paediatric Cardiology | 2013 |
| 141 | Scaphoid fracture [Skaphoidfraktur] | S1 | German Society for Trauma Surgery | 2008 |
| 142 | Descensus genitalis  [Descensus genitalis] | S1 | German Society of Gynaecology and Obstetrics, German Society of Urology | 2008 |
| 143 | Rectovaginal fistula  [Rektovaginale Fistel] | S3 | German Society for General and Visceral Surgery, German Society for Coloproctology | 2012 |
| 144 | Diagnosis and therapy of vulvar carcinoma and its precursors  [Diagnostik und Therapie des Vulvakarzinoms und seiner Vorstufen] | S2 | German Cancer Society and German Gynaecological Society | 2008 |
| 145 | Therapy of benign prostate syndrome  [Therapie des benignen Prostatasyndroms] | S2e | German Society of Urology | 2014 |
| 146 | Divertikulitis | S2k | German Society for Gastroenterology, Digestive and Metabolic Diseases, German Society for Visceral Surgery | 2013 |
| 147 | Exocrine Pancreas-Ca  [Exokrines Pancreas-Ca] | S3 | Guideline programme on oncology | 2013 |
| 148 | Therapy of inflammatory diseases of the palatine tonsils, tonsillitis  [Therapie der entzündlichen Erkrankungen der Gaumenmandeln, Tonsillitis] | S2k | German Society for Otorhinolaryngology, Head and Neck Surgery | 2015 |
| 149 | Diagnosis and therapy of hepatocellular carcinoma  [Diagnostik und Therapie des hepatozellulären Karzinoms] | S3 | Guideline programme on oncology | 2013 |
| 150 | Genital malformations  [Genitale Fehlbildungen] | S1 | German Society of Gynaecology and Obstetrics | 2010 |
| 151 | Ascites, spontaneous bacterial peritonitis, hepatorenal syndrome  [Aszites, spontan bakterielle Peritonitis, hepatorenales Syndrom] | S3 | German Society for General and Visceral Surgery | 2011 |
| 152 | Chronic pancreatitis  [Chronische Pankreatitis] | S3 | German Society for Gastroenterology, Digestive and Metabolic Diseases | 2012 |
| 153 | Surgery of Obesity  [Chirurgie der Adipositas] | S3 | German Society for General and Visceral Surgery | 2010 |
| 154 | Guideline on abdominal aortic aneurysm and iliac artery aneurysm  [Leitlinie zum Bauchaortenaneurysma und Beckenarterienaneurysma] | S2 | German Society for Vascular Surgery and Vascular Medicine | 2008 |
| 155 | Aortic Dissection  [Aortale Dissektion] | S2 | German Society for Vascular Surgery and Vascular Medicine | 2008 |
| 156 | Atraumatic adult femoral head necrosis  [Atraumatische Femurkopfnekrose Erwachsener] | S3 | German Society for Orthopaedics and Orthopaedic Surgery | 2014 |
| 157 | Undescended testis, maldescensus testis  [Hodenhochstand, Maldeszensus testis] | S2k | German Society for Paediatric Surgery | 2013 |
| 158 | Guidelines for interventional coronary therapy  [Richtlinien der interventionellen Koronartherapie] | N/A | German Society of Cardiology | 1997 |
| 159 | Guidelines on diseases of the renal arteries  [Leitlinie zu Erkrankungen der Nierenarterien] | S2 | German Society for Vascular Surgery and Vascular Medicine | 2008 |
| 160 | Lumbar radiculopathy [Lumbale Radikulopathie] | S2k | German Society of Neurology | 2012 |
| 161 | Guideline on diagnosis and therapy of pAVK [Leitlinie zur Diagnostik und Therapie der pAVK] | S3 | German Society for Angiology and Vascular Medicine | 2009 |
| 162 | Phimosis and paraphimosis  [Phimose und Paraphimose] | S1 | German Society for Paediatric Surgery | 2013 |
| 163 | Interdisciplinary guideline for diagnosis and therapy of stress incontinence in women  [Interdisziplinäre Leitlinie für Diagnostik und Therapie der Belastungsinkontinenz der Frau] | S2e | German Society of Gynaecology and Obstetrics, German Society of Urology | 2013 |
| 164 | Interdisciplinary guideline for diagnosis and therapy of endometriosis  [Interdisziplinäre Leitlinie zur Diagnostik und Therapie der Endometriose] | S2k | German Society for Gynaecology and Obstetrics, German Society for General and Visceral Surgery, German Society for Urology | 2013 |
| 165 | Diagnosis and therapy of gallstones  [Diagnostik und Therapie von Gallensteinen] | S3 | German Society for Gastroenterology, Digestive and Metabolic Diseases, German Society for Visceral Surgery | 2007 |
| 166 | Interdisciplinary guideline on early diagnosis and therapy of the different stages of prostate carcinoma  [Interdisziplinäre Leitlinie zur Früherkennung und Therapie der verschiedenen Stadien des Prostata-Carcinoms] | S3 | Guideline programme on oncology | 2014 |
| 167 | Guideline on the diagnosis, therapy and follow-up of renal cell carcinoma  [Leitlinie zur Diagnostik, Therapie und Nachsorge des Nierenzell-Carcinoms] | S3 | Guideline programme on oncology | 2015 |
| 168 | Guideline on the diagnosis, therapy and follow-up of patients with cervical carcinoma  [Leitlinie zur Diagnostik, Therapie und Nachsorge der Patientin mit Zervix-Carcinom] | S3 | Guideline programme on oncology | 2014 |
| 169 | Gastric carcinoma  [Magenkarzinom] | S3 | Guideline programme on oncology | 2012 |
| 170 | Gastrooesophageal reflux disease  [Gastroösophageale Refluxerkrankung] | S2k | German Society for Gastroenterology, Digestive and Metabolic Diseases | 2014 |
| 171 | Diagnosis and therapy of ulcerative colitis  [Diagnostik und Therapie der Colitis ulcerosa] | S3 | German Society for Gastroenterology, Digestive and Metabolic Diseases | 2011 |
| 172 | Indication and methodology of hysterectomy for benign diseases  [Indikation und Methodik der Hysterektomie bei benignen Erkrankungen] | S3 | German Society of Gynaecology and Obstetrics | 2015 |
| 173 | Breast reconstruction with autologous tissue  [Brustrekonstruktion mit Eigengewebe] | S3 | German Society of Gynaecology and Obstetrics | 2015 |
| 174 | Guideline for the management of perineal lacerations III. and IV.Degree after vaginal birth  [Leitlinie zum Management von Dammrissen III. Und IV. Grades nach vaginaler Geburt] | S1 | German Society for Gynaecology and Obstetrics | 2014 |
| 175 | Diagnosis and therapy of squamous cell carcinoma and adeno-carcinoma of the oesophagus  [Diagnostik und Therapie des Plattenepithel-Carcinoms und Adeno-Carcinoms des Ösophagus] | S3 | Guideline programme on oncology | 2015 |
| 176 | Bone cysts  [Knochenzysten] | S1 | German Society for Paediatric Surgery | 2013 |
| 177 | Aortic stenosis in children and adolescents  [Aortenisthmusstenose im Kinder- und Jugendalter] | S2k | German Society for Paediatric Cardiology | 2013 |
| 178 | Diagnosis and therapy of chronic pulmonary hypertension [Diagnostik und Therapie der chronisch-pulmonalen Hypertonie] | N/A | German Society of Cardiology | 2007 |
| 179 | Diagnostic cerebrospinal fluid puncture  [Diagnostische Liquorpunktion] | S1 | German Society of Neurology | 2012 |
| 180 | Epidural spinal cord stimulation for the therapy of chronic pain  [Epidurale Rückenmarksstimulation zur Therapie chronischer Schmerzen] | S3 | German Society for Neurosurgery, German Society for Neurology | 2013 |
| 181 | Diagnosis and therapy of extracranial carotid stenosis  [Diagnostik und Therapie der extrakraniellen Carotisstenose] | S3 | German Society of Neurology | 2012 |
| 182 | Guideline Vascular Infections  [Leitlinie Gefäßinfektionen] | N/A | German Society for Vascular Medicine and Vascular Surgery | 2008 |
| 183 | Guideline for the treatment of adults with congenital heart defects  [Leitlinie zur Behandlung von Erwachsenen mit angeborenen Herzfehlern] | N/A | German Society of Cardiology | 2008 |
| 184 | Infarct-related cardiogenic shock  [Infarktbedingter kardiogener Schock] | S3 | German Society of Cardiology | 2011 |
| 185 | Guideline on catheter ablation  [Leitlinie zur Katheterablation] | N/A | German Society of Cardiology | 2007 |
| 186 | Valve Vitiation in Adulthood  [Klappenvitien im Erwachsenenalter] | N/A | German Society of Cardiology | 2006 |
| 187 | Diagnostic cardiac catheterisation  [Diagnostische Herzkatheteruntersuchung] | N/A | German Society of Cardiology | 2008 |
| 188 | Guidelines for the diagnosis and treatment of coronary heart disease  [Leitlinie zur Diagnostik und Behandlung der koronaren Herzerkrankung] | N/A | German Society of Cardiology | 2003 |
| 189 | Normal pressure hydrocephalus  [Normaldruckhydrozephalus] | S1 | German Society of Neurology | 2012 |
| 190 | Polytrauma / Shear Injury Treatment  [Polytrauma / Scherverletzten-Behandlung] | S3 | German Trauma Society | 2011 |
| 191 | Craniocerebral Trauma Children  [Schädel-Hirn-Trauma Kinder] | S2k | German Society for Neonatology and Intensive Care Medicine, German Society for Paediatric Surgery, German Society for Neurosurgery | 2011 |
| 192 | Thoracic and thoracoabdominal aneurysms  [Thorakale und thorakoabdominelle Aneurysmen] | N/A | German Society for Vascular Medicine and Vascular Surgery | 2010 |
| 193 | Prevention, diagnosis, therapy and follow-up of lung carcinoma  [Prävention, Diagnostik, Therapie und Nachsorge des Lungenkarzinoms] | S3 | German Society for Thoracic Surgery | 2010 |
| 194 | Univentricular heart in childhood and adolescence  [Univentrikuläres Herz im Kindes- und Jugendalter] | S2k | German Society for Paediatric Cardiology | 2013 |
| 195 | Unruptured intracranial aneurysms  [Unrupturierte intrakranielle Aneurysmen] | S1 | German Society of Neurology | 2012 |
| 196 | Valvular pulmonary stenosis  [Valvuläre Pulmonalstenose] | S2k | German Society of Paediatric Cardiology | 2013 |
| 197 | Ventricular septal defect  [Ventrikelseptumdefekt] | S2k | German Society for Paediatric Cardiology | 2013 |
| 198 | Care of peripheral nerve injuries  [Versorgung peripherer Nervenverletzungen] | S3 | German Society for Neurosurgery, German Society for Neurology, German Society for Orthopaedics and Orthopaedic Surgery | 2013 |
| 199 | Diagnosis and therapy of aneurysms of the coeliac trunk, lienal, hepatic and mesenteric arteries  [Diagnostik und Therapue der Aneurysmen des Truncus coeliacus, der A. Lienalis, hepatica und mesenterica] | S2 | German Society for Vascular Medicine and Vascular Surgery | 2008 |
| 200 | Cerebral vascular formations  [Zerebrale Gefäßformationen] | S1 | German Society of Neurology | 2012 |
| 201 | Use of intra-aortic balloon counterpulsation in cardiac surgery  [Einsatz der intraaortalen Ballongegenpulsation in der Herzchirurgie] | S3 | German Society for Thoracic, Cardiac and Vascular Surgery | 2015 |
| 202 | Spontaneous dissections of the extracranial brain-supplying arteries  [Spontane Dissektionen der extrakraniellen hirnversorgenden Arterien] | S2k | German Society of Neurology | 2012 |
| 203 | Crohn's disease  [Morbus Crohn] | S3 | German Society for Gastroenterology, Digestive and Metabolic Diseases | 2014 |
| 204 | Transposition of large arteries  [Transposition großer Arterien] | S2k | German Society for Paediatric Cardiology | 2013 |
| 205 | Blunt aortic injury and traumatic aortic aneurysm  [Stumpfe Aortenverletzung und traumatisches Aortenaneurysma] | S2 | German Society for Vascular Medicine and Vascular Surgery | 2008 |
| 206 | Diseases of the femoral arteries  [Erkrankungen der Oberschenkelarterien] | S1 | German Society for Vascular Medicine and Vascular Surgery | 2008 |
| 207 | Vascular access haemodialysis  [Gefäßzugang Hämodialyse] | N/A | German Society of Nephrology, German Society of Vascular Surgery and Vascular Medicine | 2009 |
| 208 | Pulmonary arterial hypertension in childhood  [Pulmonalarterielle Hypertonie im Kindesalter] | S2k | German Society for Paediatric Cardiology | 2012 |
| 209 | Endoprosthesis coxarthrosis  [Endoprothese Coxarthrose] | S1 | German Society for Trauma Surgery | 2008 |
| 210 | Diagnosis and therapy of symptomatic ductus arteriosus of the premature infant  [Diagnostik und Therapie des symptomatischen Ductus arteriosusdes Frühgeborenen] | S2k | Society for Neonatology, Paediatric Intensive Care Medicine, German Society for Paediatric Surgery  of the German Society for Paediatrics and Adolescent Medicine | 2011 |
| 211 | VTE prophylaxis  [VTE-Prophylaxe] | S3 | 27 Medical Associations included / Intersectional Guideline | 2009 |
| 212 | Malignant melanoma  [Malignes Melanom] | S3 | Guideline programme on oncology | 2013 |
| 213 | Colorectal Ca  [Kolorektales Ca] | S3 | Guideline programme oncology | 2013 |
| 214 | Cryptoglandular anal fistula  [Kryptoglanduläre Analfisteln] | N/A | Ommer et al. | 2011 |

^1^ S1: Recommendations by committee of experts; S2: Guidelines based on evidence (S2e) or consensus of a representative committee (S2k); S3: Guidelines based on evidence and consensus of a representative committee.
