# Supplementary material for: The discussion of risk in German surgical clinical practice guidelines: a qualitative review
Source: Innov Surg Sci. 2021 Aug 25;6(2):53–7. doi: 10.1515/iss-2020-0026 (PMC8435268; doi:10.1515/iss-2020-0026)
Supplement: Supplementary file 2 [file iss-06-20200026-s002.pdf]

Reviewer Assessment

S McLennan, C Jansen and A Buyx.: The Discussion of Risk in German Surgical Clinical Practice Guidelines: A Qualitative Review

Reviewers' Comments to Original Submission

Reviewer 1: Debus, Sebastian

Date received: 18-Aug-2020
Reviewer recommendation: **Return to author for major modifications**
Reviewer overall scoring: **Medium**

Assessment Form scores: 5 = High/Yes; 3 = Medium/Adequate; 1 = Low

Is the subject area appropriate for the journal	5				
Does the title clearly reflect the paper's content?		4			
Does the abstract clearly reflect the paper's content		4			
Do the keywords clearly reflect the paper's content?					1
Does the introduction present the problem clearly?	5				
Are the results/ conclusions justified?		4			
How comprehensive and up-to-date is the subject matter presented?		4			
How adequate is the data presentation?			3		
Are units and terminology used correctly?	5				
Is the number of cases adequate?	5				
Are the experimental methods/ clinical studies adequate?		4			
Is the length appropriate in relation to the content?		4			
Does the reader get new insights from the article?		4			
Please rate the practical significance.	5				
Please rate the accuracy of methods.		4			
Please rate the statistical evaluation and quality control.		4			
Please rate the appropriateness of the figures and tables.		4			
Please rate the appropriateness of the references.		4			
Please evaluate the writing style and use of language.		4			
Please judge the overall scientific quality of the manuscript.			3		
Are the methods used worthy of reproduction in greater deal?	yes				
Would you be willing to review a revision of this manuscript?	yes				

Comments to author: This is a topic of high value and should be published.
Search terms are not mentioned: was it just "risk" and "complication"?

It should be stated, how many CPGs from which surgical specialty have been included in the analysis
Did the authors search for risk-benefit ratios in the guidelines? This would be essential, especially when discussing ethical issues. Please discuss.
Did the authors look into international guidelines? Is there a more distinct focus on surgical risk?
The authors mention the guidelines from the German Society for Cardiology, as the sole CPG performed outside AWMF standards. But they do not discuss possible differences in the discussion. This should be added.

Reviewer 2: Sauerland, Stefan
Date received: 24-Jan-2021
Reviewer recommendation: **Return to author for minor modifications**
Reviewer overall scoring: **High**

Assessment Form scores: 5 = High/Yes; 3 = Medium/Adequate; 1 = Low

Is the subject area appropriate for the journal	5				
Does the title clearly reflect the paper's content?		4			
Does the abstract clearly reflect the paper's content		4			
Do the keywords clearly reflect the paper's content?					1
Does the introduction present the problem clearly?		4			
Are the results/ conclusions justified?			3		
How comprehensive and up-to-date is the subject matter presented?		4			
How adequate is the data presentation?		4			
Are units and terminology used correctly?	5				
Is the number of cases adequate?	5				
Are the experimental methods/ clinical studies adequate?		4			
Is the length appropriate in relation to the content?		4			
Does the reader get new insights from the article?		4			
Please rate the practical significance.		4			
Please rate the accuracy of methods.		4			
Please rate the statistical evaluation and quality control.		4			
Please rate the appropriateness of the figures and tables.			3		
Please rate the appropriateness of the references.		4			
Please evaluate the writing style and use of language.		4			
Please judge the overall scientific quality of the manuscript.		4			
Are the methods used worthy of reproduction in greater deal?	No				
Would you be willing to review a revision of this manuscript?	Yes				

Comments to author: This article presents an interesting analysis of clinical practice guidelines (CPGs). The topic of surgical risk communication is innovative and important and the methods employed by the authors are good. However, the conclusions appear to be somewhat misguided by over expectations.

1. It certainly would be desirable, if CPGs would contain specific results for all key outcomes of all interventions. For a balanced view, not only risks but also potential benefits would have to be reported in absolute numbers. So far, CPGs focus on relative rather than absolute effects, e.g. by stating that intervention A as compared to interventions B reduces the risk of an event by 50%. If guideline authors would be forced to quantify complication risks, they would take the required numbers

from the randomized evidence that was already used for estimating relative effectiveness. As a result, the incidence of complications would be under- estimated (or overestimated), because clinical trials usually exclude higher (or lower) risk patients. Therefore, guideline authors would have to perform additional searches for observational studies – ideally based on routine data from Germany. In my view, it would not be wise to drown guideline authors in even more work. Relative estimates of risk are usually more important than absolute estimates.

2. The authors call for "evidence-based thresholds" regarding the classification of high risk or low risk. However, questions of definition cannot be answered in an evidence-based way. What constitutes "high risk" requires an intuitive definition set by an authoritative body. In surgery, for example, many groups have adopted a simple scheme, where low, medium and high-risk surgery are defined to have <1%, 1 to 5%, or >5% mortality (within 30 days after the intervention), respectively.

3. For an "in-depth consideration of specific, procedure-related risk" (p.6) it would be necessary not only to describe the average risk, but also to estimate the variation in risk and the factors that are associated with higher or lower risk. Specific or even individualized estimation and communication of risk is highly important, but again should not be the task of guideline authors.

4. Unfortunately, the selected 214 CPGs are not described with regard to their methodology. As a basic requirement, guideline quality should be reported in the manuscript by providing the number of guidelines that were evidence- and consensus-based (S3 in the German classification), evidence-based (S2e), consensus-based (S2k), or neither evidence- nor consensus-based (S1). It then would be very useful to cross-tabulate guideline quality levels and quality of risk information. If higher guideline quality is associated with better information on risk, one could conclude that the current striving for higher-quality CPGs will probably be sufficient to achieve also better risk information within these CPGs. Specifically, the use of GRADE methodology (including summary of findings tables) certainly would help, as reporting of numerical data on key outcomes is standard when using this methodology for CPG development.

5. In the discussion (p.6), valid and transparent information on risks is described as a prerequisite for the patient´s right to make well-informed autonomous choices. In spite of the importance of these aims, health professionals usually are relying on multiple sources of medical information (e.g. textbooks, primary studies, quality control data, own experience, etc.). Therefore, the statement that CPGs without detailed information on risks might "violate" the patient's rights appears to be overemphasized.

6. It remains unclear, what "numerical risk thresholds" exactly are. First, risk thresholds could be used to differentiate low, medium and high risks (see my comment No. 2). Secondly, however, risk thresholds could be used to guide individual decision-making by balancing risks and benefits. For example, guideline authors could argue that patients with a symptomatic high-grade stenosis of the carotid artery have a five-year risk of stroke in the range of 10%, which in turn renders carotid endarterectomy acceptable, if the periprocedural risk of stroke is below 2%. These considerations again highlight that risk alone is an insufficient basis for deciding on surgical indications.

7. In the discussion, please correct "CPGs are an importance source" to "… an important source".

8. Tables 2, 3, 4 and 5 could all be condensed into just one table. Maybe, even Table 1 might be added to the new table.

Authors' Response to Reviewer Comments

Date received: 08-Mar-2021

Response to reviewer 1

Comments to the Author

This is a topic of high value and should be published.
Response: Thank you for your positive assessment of our research.

Search terms are not mentioned: was it just "risk" and "complication"?
Response: In the manuscript under data collection we state:
"In November 2015, all CPGs publicly available on the website of the German umbrella organization of medical professional associations (AWMF) were collected by CJ. In addition, GPGs on invasive cardiology were collected from the website of the German Association for Cardiology (DGK), because guidelines for invasive cardiology procedures (e.g. cardiac catheterization) are specifically not covered by the AWMF. Based on the inclusion criteria, CJ screened the CPGs in order to assess for eligibility for inclusion in the review. In case of uncertainty, the CPG was discussed with project members."

Search terms were not used in data collection; all publicly available CPGs were collected from these websites to have a complete/full sample. The guidelines were then screened based on the inclusion criteria. The terms "risk" and "complication" were not part of the inclusion criteria because we wanted to examine "the content of German surgical CPGs in relation to surgical risks". As we show in the results section, "5% (10/214) of guidelines did not discuss "risks" or "complications" at all."

It should be stated, how many CPGs from which surgical specialty have been included in the analysis
Response: In the first paragraph of the results section it is stated "The included CPGs were published by a total of 11 different specialties, however, 73.3% (157/214) of included GPGs came from fives specialties (paediatric surgery, neurosurgery, trauma and orthopaedic surgery, vascular surgery, general and visceral surgery) (see Table 1). Full information can be found in table 1 of the manuscript:

**Specialty**	**Number of guidelines n/N (%)**
Paediatric Surgery	58/214 (27.1)
Neurosurgery, including invasive Neurology	29/214 (13.5)
Trauma and Orthopaedic Surgery	26/214 (12.1)
Vascular Surgery	25/214 (11.7)
General and Visceral Surgery	19/214 (8.9)
Obstetrics and gynaecology	15/214 (7.0)
Interdisciplinary Guidelines & Collaborations	14/214 (6.5)
Cardiothoracic Surgery, including invasive Cardiology	13/214 (6.1)
Ear Nose Throat	8/214 (3.7)
Dermatology	6/214 (2.8)
Urology	1/214 (0.5)

Did the authors search for risk-benefit ratios in the guidelines? This would be essential, especially when discussing ethical issues. Please discuss
Response: Benefits of the various interventions and within disciplines are discussed in great length in the literature and in the guidelines. It was our hypothesis that risks are not discussed similarly. Therefore, we focused on risks and harms in this work. Our hypothesis was confirmed. A next step would indeed be to evaluate risk-benefit ratios and we take this suggestion on board for our future work and analyses.

Did the authors look into international guidelines? is there a more distinct focus on surgical risk?
The authors mention the guidelines from the German Society for Cardiology, as the sole CPG performed outside AWMF standards. But they dont discuss possible differences in the discussion. This should be added.

Response: Thank you, the international context is certainly relevant and in our introduction we discuss so of the debate from the international literature. However, regarding our empirical data, we wanted to focus on Germany. It is therefore noted in the limitations section that "only CPGs from Germany were included in the review. Risk assessment in CPGs in other countries might differ significantly from our sample. However, the finding that a substantial number of German CPGs do not provide specific guidance regarding the risk assessment of surgical complications can help highlight the importance of this issue internationally."
We understand that AWMF is the most comprehensive database of German CPGs, and was there selected as the main database for data collection. However, it was clear that when it came to guidelines relating to heart disease, only find pediatric cardiology and cardiothoracic surgery CPGs could be found via the AWMF. The German Society of Cardiology - publishes their own CPGs after a specific evaluation process and then publishes them in their specialty journal ("Der Kardiologe"). From what we can gather, the process itself is not different from that of other specialties, they only seem to publish the CPGs independently. We had noted this in the data collection section of the methods.

Response to reviewer 2

This article presents an interesting analysis of clinical practice guidelines (CPGs). The topic of surgical risk communication is innovative and important and the methods employed by the authors are good. However, the conclusions appear to be somewhat misguided by over expectations.
1. It certainly would be desirable, if CPGs would contain specific results for all key outcomes of all interventions. For a balanced view, not only risks but also potential benefits would have to be reported in absolute numbers. So far, CPGs focus on relative rather than absolute effects, e.g. by stating that intervention A as compared to interventions B reduces the risk of an event by 50%. If guideline authors would be forced to quantify complication risks, they would take the required numbers from the randomized evidence that was already used for estimating relative effectiveness. As a result, the incidence of complications would be under- estimated (or overestimated), because clinical trials usually exclude higher (or lower) risk patients. Therefore, guideline authors would have to perform additional searches for observational studies – ideally based on routine data from Germany. In my view, it would not be wise to drown guideline authors in even more work. Relative estimates of risk are usually more important than absolute estimates.
Response: Thank you for this very interesting consideration. The aim of our paper is mainly to point out the lack of inclusion of risk in some of the available guidelines and to encourage that risk is considered more explicitly when they are written. However, we leave it largely open how exactly that should happen. Indeed, we think it best to leave it to those developing guidelines how to adopt our suggestions and put them into practice while considering aspects such as the ones that you have raised.

2. The authors call for "evidence-based thresholds" regarding the classification of high risk or low risk. However, questions of definition cannot be answered in an evidence-based way. What constitutes "high risk" requires an intuitive definition set by an authoritative body. In surgery, for example, many groups have adopted a simple scheme, where low, medium and high-risk surgery are defined to have <1%, 1 to 5%, or >5% mortality (within 30 days after the intervention), respectively.
Response: We are not sure we understand this comment. We assumed that the schemes and thresholds adopted in the field are based on evidence. Looking at the specific types of risks we discuss in this article, our recommendations include that the definition of future thresholds should lie with the experts in the field.

3. For an "in-depth consideration of specific, procedure-related risk" (p.6) it would be necessary not only to describe the average risk, but also to estimate the variation in risk and the factors that are associated with higher or lower risk. Specific or even individualized estimation and communication of risk is highly important, but again should not be the task of guideline authors.

Response: Again, this is an interesting comment. However, the recommendations of this paper have a more modest aim, namely to encourage exactly this kind of debate in the field, amongst the experts writing guidelines. We are not well placed to discuss this in detail.

4. Unfortunately, the selected 214 CPGs are not described with regard to their methodology. As a basic requirement, guideline quality should be reported in the manuscript by providing the number of guidelines that were evidence- and consensus-based (S3 in the German classification), evidence-based (S2e), consensus-based (S2k), or neither evidence- nor consensus-based (S1). It then would be very useful to cross-tabulate guideline quality levels and quality of risk information. If higher guideline quality is associated with better information on risk, one could conclude that the current striving for higher-quality CPGs will probably be sufficient to achieve also better risk information within these CPGs. Specifically, the use of GRADE methodology (including summary of findings tables) certainly would help, as reporting of numerical data on key outcomes is standard when using this methodology for CPG development.
Response: Thank you. A table has been added that lists all included CPGs by risk group, including CPG title, quality category (S1-S3), author, and year updated.

5. In the discussion (p.6), valid and transparent information on risks is described as a prerequisite for the patient´s right to make well-informed autonomous choices. In spite of the importance of these aims, health professionals usually are relying on multiple sources of medical information (e.g. textbooks, primary studies, quality control data, own experience, etc.). Therefore, the statement that CPGs without detailed information on risks might "violate" the patient's rights appears to be overemphasized.
Response: Thank you. We believe that insufficient information about the risk of procedures can potentially undermine informed consent and thus patient autonomy. It is also established terminology to say that some things "violate" ethical principles. However, to avoid the impression of overemphasis, we have rephrased the sentence and use more moderate language now.

6. It remains unclear, what "numerical risk thresholds" exactly are. First, risk thresholds could be used to differentiate low, medium and high risks (see my comment No. 2). Secondly, however, risk thresholds could be used to guide individual decision-making by balancing risks and benefits. For example, guideline authors could argue that patients with a symptomatic high-grade stenosis of the carotid artery have a five-year risk of stroke in the range of 10%, which in turn renders carotid endarterectomy acceptable, if the periprocedural risk of stroke is below 2%. These considerations again highlight that risk alone is an insufficient basis for deciding on surgical indications.
Response: Thank you for this comment. We believe there might be a misunderstanding here. Our data and paper points out that risks should be considered more explicitly in future guidance construction (we do not make suggestions for individual, patient-level decision-making in this paper). However, nowhere in the paper do we say that risk alone is a sufficient basis for deciding on surgical indications. We would not agree with that at all and have added a sentence towards the end to make sure that this cannot be misread. We hope this is clearer now.

7. In the discussion, please correct "CPGs are an importance source" to "... an important source".
Response: This has been corrected, thank you for bringing this mistake to our attention.

8. Tables 2, 3, 4 and 5 could all be condensed into just one table. Maybe, even Table 1 might be added to the new table.
Response: The tables have been combined into one table. Thank you for the suggestion.

Reviewers' Comments to Revised Submission

Reviewer 1: Debus, Sebastian

Date received: 19-Mar-2021

Reviewer recommendation: **Accept in present form**
Reviewer overall scoring: **High**

Assessment Form scores: 5 = High/Yes; 3 = Medium/Adequate; 1 = Low

Is the subject area appropriate for the journal	5				
Does the title clearly reflect the paper's content?	5				
Does the abstract clearly reflect the paper's content	5				
Do the keywords clearly reflect the paper's content?	5				
Does the introduction present the problem clearly?	5				
Are the results/ conclusions justified?		4			
How comprehensive and up-to-date is the subject matter presented?		4			
How adequate is the data presentation?		4			
Are units and terminology used correctly?		4			
Is the number of cases adequate?		4			
Are the experimental methods/ clinical studies adequate?		4			
Is the length appropriate in relation to the content?		4			
Does the reader get new insights from the article?		4			
Please rate the practical significance.		4			
Please rate the accuracy of methods.		4			
Please rate the statistical evaluation and quality control.		4			
Please rate the appropriateness of the figures and tables.	5				
Please rate the appropriateness of the references.	5				
Please evaluate the writing style and use of language.	5				
Please judge the overall scientific quality of the manuscript.	5				
Are the methods used worthy of reproduction in greater deal?	Yes				
Would you be willing to review a revision of this manuscript?	Yes				

Comments to author: Thank you for revising your primary manuscript. You have addressed all relevant comments from the reviewers adequately and comprehensive. This new version of the manuscript reads very well and i see no additional need for further revision.

Reviewer 1: Sauerland, Stefan

Date received: 12-Mar-2021
Reviewer recommendation: **Return to author for minor modifications**
Reviewer overall scoring: **Medium**

Assessment Form scores: 5 = High/Yes; 3 = Medium/Adequate; 1 = Low

Is the subject area appropriate for the journal		4			
Does the title clearly reflect the paper's content?		4			
Does the abstract clearly reflect the paper's content		4			

Do the keywords clearly reflect the paper's content?		4			
Does the introduction present the problem clearly?		4			
Are the results/ conclusions justified?		4			
How comprehensive and up-to-date is the subject matter presented?		4			
How adequate is the data presentation?			3		
Are units and terminology used correctly?		4			
Is the number of cases adequate?	5				
Are the experimental methods/ clinical studies adequate?		4			
Is the length appropriate in relation to the content?	5				
Does the reader get new insights from the article?		4			
Please rate the practical significance.			3		
Please rate the accuracy of methods.		4			
Please rate the statistical evaluation and quality control.			3		
Please rate the appropriateness of the figures and tables.				2	
Please rate the appropriateness of the references.		4			
Please evaluate the writing style and use of language.		4			
Please judge the overall scientific quality of the manuscript.		4			
Are the methods used worthy of reproduction in greater deal?	No				
Would you be willing to review a revision of this manuscript?	Yes				

Comments to author: The reviewer (#2) thanks the authors for responding to the original comments. Most have been resolved, but I want to comment on two remaining issues, that I believe are not fully resolved.
In my eyes, it is still unclear, what "numerical risk thresholds" are, and I apologize for being so stubborn (previous comments #2 and #6). The word 'threshold' means the level at which something starts (or stops), or should start (or stop). To me, however, it is unclear what consequences are implied, when a guideline provides numerical risk thresholds. If for example a guideline describes, that a recurrence rate of under 5% is a common results (or a useful threshold) after the resection of early stage gastric cancer, what does this mean? In a given patient, should I refrain from surgery, if the individual recurrence risk is estimated to be over 5%, or should the hospital reconsider its treatment pathways and quality, if recurrence risk is over 5% on average? I simply don't understand what the authors mean, when reporting that "39% (84/214) of guidelines [...] provided numerical risk thresholds". Perhaps these are not thresholds, but rather numerical specifications or estimates only?
In my previous comment #4, I had proposed "to cross-tabulate guideline quality levels and quality of risk information". The new table that was newly added is clearly inadequate, because it is much too long and publishable only as an online appendix. Therefore, I prepared the table by counting all categories. This new table clearly shows that higher quality of risk description in the guidelines is strongly associated with methodological guideline quality. I propose that the authors use this table instead of their very long table.

Authors' Response to Reviewer Comments

Date received: 23-Mar-2021

Response to reviewer 1

Thank you for revising your primary manuscript. You have addressed all relevant comments from the reviewers adequately and comprehensive. This new version of the manuscript reads very well and i see no additional need for further revision.

Response: Many thanks for your comments and positive feedback.

Response to reviewer 2

The reviewer (#2) thanks the authors for responding to the original comments. Most have been resolved, but I want to comment on two remaining issues, that I believe are not fully resolved.
In my eyes, it is still unclear, what “numerical risk thresholds” are, and I apologize for being so stubborn (previous comments #2 and #6). The word ‘threshold’ means the level at which something starts (or stops), or should start (or stop). To me, however, it is unclear what consequences are implied, when a guideline provides numerical risk thresholds. If for example a guideline describes, that a recurrence rate of under 5% is a common results (or a useful threshold) after the resection of early stage gastric cancer, what does this mean? In a given patient, should I refrain from surgery, if the individual recurrence risk is estimated to be over 5%, or should the hospital reconsider its treatment pathways and quality, if recurrence risk is over 5% on average? I simply don’t understand what the authors mean, when reporting that “39% (84/214) of guidelines [...] provided numerical risk thresholds”. Perhaps these are not thresholds, but rather numerical specifications or estimates only?
Response: Thank you for following up regarding this matter. Yes, we agree that the term “thresholds” could be unclear for the reasons outlined by the reviewer. We have replaced the word “thresholds” with “estimates” for clarification.

In my previous comment #4, I had proposed “to cross-tabulate guideline quality levels and quality of risk information”. The new table that was newly added is clearly inadequate, because it is much too long and publishable only as an online appendix. Therefore, I prepared the table by counting all categories. This new table clearly shows that higher quality of risk description in the guidelines is strongly associated with methodological guideline quality. I propose that the authors use this table instead of their very long table.
Response: Thank you for this suggestion. We agree that it would be best to put the long table listing all guidelines in a supplementary table, and add a table summarising risk categories and quality categories. We have not received the table the reviewer refers to, but have created our own.

Comments by the Editor-in-Chief to Revised Submission

Since the first reviewer recommended to accept the manuscript and all comments from the 2nd reviewer were adequately dealt with, I accept the revised manuscript.
